# Comparative Evaluation of Linscope and King Vision Video Laryngoscopes in Tracheal Intubation: A Randomized Controlled Trial

**DOI:** 10.7759/cureus.56514

**Published:** 2024-03-19

**Authors:** Mohd Luqman, S. Moied Ahmed, Amal Shaharyar, Raihan Mannan

**Affiliations:** 1 Cardiac Anesthesia, Fortis Hospital Mohali, Mohali, IND; 2 Anesthesia and Critical Care, Jawaharlal Nehru Medical College and Hospital, Aligarh Muslim University, Aligarh, IND; 3 Endocrinology, Postgraduate Institute of Medical Education and Research, Chandigarh, IND; 4 Physiology, All India Institute of Medical Sciences Patna, Patna, IND

**Keywords:** video laryngoscopy (vl), randomized controlled trial (rct), tracheal intubation, king vision video laryngoscope, linscope video layngoscope

## Abstract

Introduction

Indirect laryngoscopy has become a widely accepted method for learning the techniques of airway management. The incorporation of small, less expensive, and yet more reliable video cameras in laryngoscopes has given the process of laryngoscopy and intubation a big leap. The King Vision video laryngoscope (Medline Industries, USA) has demonstrated promise in several settings while the Linscope video laryngoscope (Centrum, Turkey) is a newly launched device and no literature is available to the best of our knowledge. Therefore, we aimed to compare the performance of the Linscope video laryngoscope (VL) and King Vision video laryngoscope.

Method

This is a randomized controlled trial study. Seventy patients, after approval from the institute's ethical clearance, were divided into two groups. In Group A, patients were intubated with Linscope VL and in Group B patients were intubated with King Vision VL as per the protocol. The primary outcome measure was the duration of tracheal intubation. Secondary outcomes were measured by the number of attempts, ease of intubation, and glottic view.

Results

Both Linscope VL and King Vision VL groups were comparable in terms of mean intubation time (20.34 s vs. 19.45 s). The endotracheal intubation with both devices was 100% successful at the first attempt. Both the devices provided a percentage of glottic opening (POGO) score of > 70% and a clear vision of the glottis. The POGO score obtained with King Vision VL was 83.57 ± 11.41% and with Linscope VL was 87.85 ± 10.31%. POGO score was greater with Linscope VL compared to King Vision VL, but the difference was not statistically significant (p-value>0.05).

Conclusion

King Vision demonstrated shorter intubation time and fewer optimization maneuvers. Both devices achieved a 100% success rate on the first attempt. While both devices are viable first-line options, King Vision's well-established efficacy in the literature suggests its preference over Linscope till extensive evidence is available in the future.

## Introduction

Endotracheal intubation is a specialised learned skill, and difficult airway remains an important risk factor for patient outcomes [1]. Video laryngoscopes (VLs) have shown promising results in managing difficult airways. Nowadays a variety of VLs are available in the market. So it becomes crucial to choose a VL that will be useful in more difficult laryngoscopy and intubation. In addition, it should have a high success rate of intubation, require less adjustment in maneuver, technique that mimics conventional laryngoscopy, can be reused.

We have taken a new video laryngoscope i.e. Linscope VL (Centrum, Turkey) which has a detachable and disposable channeled blade and can be attached to handle having a screen. The screen can be connected to a projector for display. As the Linscope VL is a recently introduced device, its efficacy as an intubating tool needs assessment compared to the commonly used and extensively studied video laryngoscope. The Airtraq VL also has an extensive body of literature supporting its application in difficult airways [2-4]. It has been studied in patients at both lower risk [5] and higher risk [6-8] for difficult tracheal intubation [9].

Because of the operational differences, we hypothesised that Linscope VL reduces intubation difficulty in comparison with King Vision VL (Medline Industries, USA). Therefore, we aimed to compare the effectiveness of the Linscope video laryngoscope and King Vision video laryngoscope as an intubation aid.

## Materials and methods

Following approval by the Institutional Ethical Committee (approval no. 191IEC, JNMCH), the study was conducted at Jawaharlal Nehru Medical College and Hospital, Aligarh Muslim University, Aligarh, India. Seventy patients who were undergoing elective general surgery under general anesthesia were recruited for the study. All patients had thorough pre-anesthetic checkups and those meeting the criteria were included in the study. Written informed consent was obtained from patients. According to the American Society of Anesthesiologists (ASA) physical status classification system, ASA Grade I (patient is a completely healthy fit patient) and Grade II (patient has mild systemic disease) were eligible for inclusion in the study. Both male and female patients aged 20-65 years, weighing between 40-75 kg, with a BMI ≤ 30 kg/m^2^, and representing all classes of Mallampati (MP), were included. Exclusion criteria encompassed patients with a history of multiple/failed intubation, predicted difficult laryngoscopy, oral pathology obstructing device insertion, mouth opening <2.5 cm, conditions associated with a potentially full stomach (such as trauma, morbid obesity, or pregnancy), and risks of gastroesophageal reflux or hiatus hernia. The trial was registered with the Clinical Trials Registry of India on July 29, 2019 (reference number: CTRI/2019/07/020427).

Randomisation of patients

**Figure 1:**
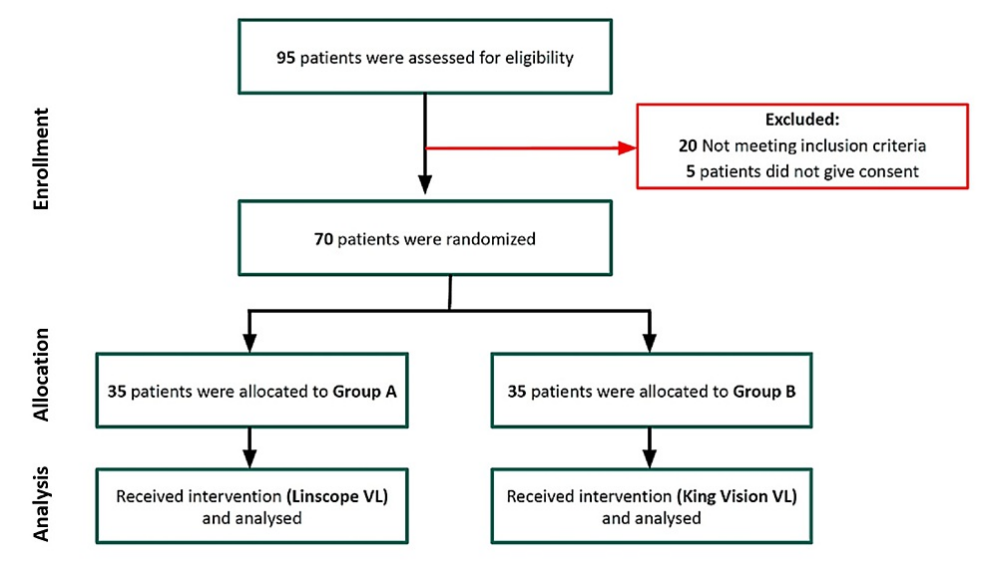
Consolidated Standards of Reporting Trials (CONSORT) diagram.

The patients were assigned randomly to two groups using computer-generated random numbers. The allocation details were concealed within sealed envelopes, which remained unopened until patients provided their consent. Patients of Group A (n=35, study group) were intubated using a Linscope video laryngoscope. Patients of Group B (n=35, control group) were intubated using a King Vision video laryngoscope (Figure 1). If intubation was not achieved, the patient was declared failed intubation and the airway was managed with a second-generation supraglottic airway device (SAD).

Sample size calculation

The sample size was calculated using the formula:

$n = \frac{2\times (Z_{1-\alpha /2} + Z_{1-\beta} )^{2}\times \sigma^{2}}{d^{2}}$

Where d = difference in intubation time between the two devices less than 10 seconds; σ = pooled standard deviation which is 12 as derived from previous studies; Z_1−α/2_ = 1.96 is a standard normal value at a 5% level of significance; and Z_1−β_ = 1.28 is a standard normal value of 90% power. 

The minimum number of samples was 31 in each group. However, to have a margin of safety and round the figure 35 patients in each group were included for the study.

Intubation of patients

A standardized anesthesia procedure was adhered to, involving a consistent premedication regimen comprising midazolam injection at 0.03 mg/kg, ondansetron at 0.10 mg/kg, and fentanyl at 1 μg/kg. In the operating room, patients underwent continuous monitoring of ECG, pulse rate, SpO_2_, non-invasive blood pressure (NIBP), and end-tidal carbon dioxide (EtCO_2_) using a multi-channel monitor (Nihon Kohden, Tokyo, Japan). After pre-oxygenation, anesthesia induction was performed with a propofol injection (2-2.5 mg/kg), followed by achieving neuromuscular blockade through a suxamethonium injection (1-2 mg/kg).

The patients were then intubated with either a Linscope video laryngoscope (Group A) or King Vision video laryngoscope (Group B) according to the allocated group. They were initially intubated in a neutral position with or without optical external laryngeal manipulation (OELM) or airway adjuncts (stylet). If failed, then intubation was done in the sniffing position with or without OELM or airway adjuncts. The procedure ended after confirmation of endotracheal tube placement by capnography.

Failed intubation was considered if the trachea was not intubated after a maximum of three attempts and with all adjusting maneuvers i.e., sniffing position, OELM, and airway adjunct. The technique was abandoned, and a second-generation supraglottic airway device (SAD) was inserted as a rescue device and the case was undertaken. Intubation time was defined as the duration between inserting the blade between the teeth and the placement of the endotracheal tube through the vocal cords. The time was measured in seconds by an assistant using a stopwatch.

Device description

The King Vision video laryngoscope (Medline Industries, USA) features a two-piece design with a reusable monitor and disposable channeled blade options, providing clear visualization with light-emitting diode (LED) light and complementary metal-oxide semiconductor (CMOS) camera technology. It includes an anti-fog coating for uninterrupted imaging. King Vision video laryngoscope has been routinely used in our department to manage predicted difficult airways.

**Figure 2:**
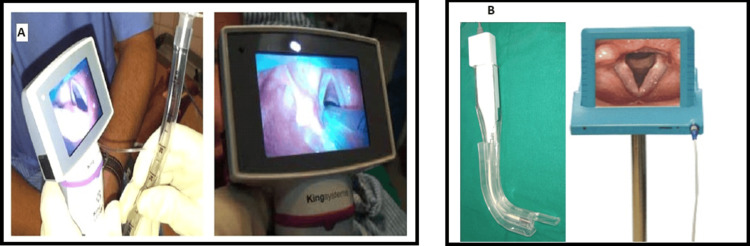
Video laryngoscopes used in the study (A) King Vision video laryngoscope (Medline Industries, USA); (B) Linscope video laryngoscope (Centrum, Turkey).

The Linscope video laryngoscope (Centrum, Turkey) integrates a 4.3 mm 1/16 CMOS intubation camera with an 8-inch LCD monitor, offering adjustable contrast, brightness, and color. Its rounded blade edge minimizes tissue injury, and the device allows video recording and image storage via a Secure Digital (SD) memory card, which is suitable for teaching purposes. The Linscope VL was procured by the Department of Anaesthesia at AMU Aligarh specifically for this study. King Vision VL has been routinely used in our department for managing predicted difficult airways (Figure 2).

Training of anesthesiologist

The measures were taken to avoid observer variability by having the same researcher perform all intubations. The anesthesiologist involved was a newly joined Resident Doctor who underwent rigorous training with both the Linscope and King Vision video laryngoscopes simultaneously. This training included 20 intubations on manikins with each device and an additional 10 intubations on different patients for both VLs. We believe this training protocol adequately prepared the anesthesiologist for the study procedures, minimizing the risk of bias. The observer recorded the total number of intubation attempts and the intubation time. He also recorded any events that occurred during intubation, such as lip or dental injury.

Assessment of difficult airway

In our assessment of difficult airways, we employed different tools to evaluate various aspects. While the Cormack-Lehane (CL) classification is commonly utilized to characterize laryngeal views during direct laryngoscopy, POGO scoring is specifically designed to assess glottic views during video laryngoscopy. In our study, we utilized Mallampati classification and POGO scoring to gauge the difficulty level of airways for intubation [10]. Additionally, we graded the ease of tracheal intubation using optical external laryngeal manipulation (OELM), as illustrated in Table 1 below.

**Table 1:** Classification and grading for assessment of difficulty level of the airway for intubation

Mallampati classification	
Class I	soft palate, uvula, and pillars are visible
Class II	soft palate and uvula are visible
Class III	only the soft palate and base of the uvula are visible
POGO scoring (Percentage of glottic opening)	
0%	When no glottis structures were visible (not even arytenoids)
33%	Only the lower 1/3rd of the vocal cords and arytenoids were visible
100%	When the entire glottis aperture was visualised
Ease of tracheal intubation grading	
Grade 1	No extrinsic manipulation of the larynx was required
Grade 2	External manipulation of the larynx was required to intubate
Grade 3	Failed intubation

Statistical analysis 

The statistical analysis was conducted utilizing IBM SPSS version 20.0 software (IBM Corp., Armonk, NY). The data are expressed in numerical figures, mean, standard deviation, and percentages as deemed appropriate. Demographic variances between the groups underwent analysis through the Chi-square and unpaired t-test. The time taken for intubation was scrutinized using an unpaired t-test, while the number of attempts and ease of intubation were assessed employing the Chi-square test. Significance in all statistical analyses was determined by a P-value < 0.05.

## Results

In this randomized controlled trial, 70 patients were included in our study; 35 each in Group A and Group B. The mean age of Group A patients was 34.00 ± 8.68 and Group B patients was 31.05 ± 8.41. The male: female ratio was 13:22 in Group A and 15:20 in Group B. There were no significant differences in patient age, BMI, and preoperative airway assessment of patients between both groups (Table 2).

**Table 2:** Demographic profile of the patients in both groups VL, video laryngoscope; M, male; F, female; BMI, body mass index; ASA, American Society of Anesthesiologists Classification

Parameter	Group A (Linscope VL)	Group B (King Vision VL)	P-value
Age (years, Mean ± SD)	34.00 ± 8.68	31.05 ± 8.41	NS
Sex (M:F)	13:22	15:20	NS
BMI (kg/m^2^, Mean± SD)	23.70 ± 3.18	24.92 ± 3.49	NS
Mallampatti class			
I	15	14	NS
II	19	19	NS
III	01	02	NS
ASA GRADE			
I	18	24	NS
II	12	06	NS

The observed ease of intubation was either grade 1 or 2 in all patients for both groups. The mean time for successful intubation was comparable in the Linscope VL group and the King Vision VL group (20.34 ± 3.90 vs 19.45 ± 2.42, p-value >0.05). The overall success rate on the first attempt was 100%, in both groups. No failure of intubation in any group. In the Linscope VL group, 82.9% of patients needed a single adjustment in maneuver and 5.7% of patients needed two adjustments in maneuver, while in the King Vision VL group, 22.9% of patients didn’t need any adjustment, 77.1% of patients needed a single adjustment in maneuver and none of the patients needed two adjustments in maneuver. The majority of the patients were categorised as a Grade I ease of intubation in both groups (57.1% in the Linscope VL group, 80% in the King Vision VL group). Analysis of data revealed that there was no episode of desaturation <90% in any of the groups (Table 3). There were no airway injuries detected in any patient in either group.

**Table 3:** Comparison of intubation parameters of the two groups VL, video laryngoscope; NS, not significant; POGO, percentage of glottic opening score

Parameters	Linscope VL (n=35)	King Vision VL (n=35)	P-value
Successful intubation	100%	100%	
Number of attempts			
First	100%	100%	
Intubation time (seconds)	20.34 ± 3.90	19.45 ± 2.42	NS
Number of adjustments in maneuver			
0	0 (0%)	8 (22.9%)	<0.01
1	29 (82.9%)	27 (77.1%)	
2	6 (5.7%)	0 (0%)	
POGO score			
>90	11 (31.4%)	9 (25.7%)	NS
<90	24 (68.6%)	26 (74.3%)	
Ease of intubation			
Grade I	20 (57.1%)	28 (80%)	NS
Grade II	15 (42.9%)	7 (20%)	
Episodes of oxygen saturation <90%	0	0	

## Discussion

In this randomized controlled trial, we conducted a comparative evaluation of the Linscope VL and King Vision VL in patients undergoing tracheal intubation. The demographic characteristics of patients in both groups were similar, ensuring that these variables did not influence our study outcomes. However, various factors such as airway anatomy, head position, preoperative anesthesia, administered drugs, and operator proficiency could potentially impact our results.

The efficacy and safety of video laryngoscopes (VLs) for tracheal intubation remain subjects of ongoing investigation, with outcomes varying depending on the specific airway conditions. A recent meta-analysis of randomized controlled trials assessed several VLs, including Airtraq, Airwayscope, C-MAC, C-MAC D-blade (CMD), GlideScope, King Vision, and McGrath, in patients undergoing general anesthesia. Among the evaluated devices, CMD and King Vision exhibited the highest rates of first-pass intubation success, with King Vision showing particular effectiveness in patients with normal airways. The meta-analysis also suggested that VLs, with the exception of McGrath, generally outperformed the Macintosh laryngoscope in terms of efficacy and safety [11].

In previous studies, the King Vision VL has demonstrated superior performance compared to other devices such as the Airway Scope, McGrath, McCoy, and Lightwand [2-7]. For instance, in a manikin-based study conducted with novice personnel, the King Vision VL's channeled blade facilitated intubation without incidence of esophageal intubation when compared to the Macintosh laryngoscope [2].

In our study, we observed that the King Vision VL exhibited shorter intubation times compared to the Linscope VL. The likely reason for this discrepancy in intubation times between the two groups could be attributed to better hand-eye coordination while using the King Vision VL, particularly with the aid of the video display screen attached to the handle. Additionally, the curvature of the King Vision VL blade closely matches the primary oropharyngeal curve, requiring less manipulation during intubation compared to the Linscope VL. Conversely, the Linscope VL's blade has a more acute distal angulation, which may result in the endotracheal tube direction missing the glottis after exiting from the blade, necessitating frequent adjustments.

The incidence of first attempts of intubation was 100% in both the King Vision VL and Linscope VL groups. Previous studies have consistently reported a high success rate with the King Vision VL compared to other VLs [4,8-10]. For instance, in a study comparing King Vision VL and Airtraq, the time required for intubation and the number of attempts were significantly shorter with King Vision VL than with Airtraq [8]. However, we found that the success rate of intubation was equally high with Linscope VL, comparable to King Vision VL. One possible reason for this observation could be that our study recruited patients with Mallampati class I and II only.

The Linscope VL group exhibited a higher incidence of adjustment maneuvers compared to the King Vision VL group. Specifically, the occurrence of a single adjustment maneuver, such as OELM or the use of external aids like stylet/bougie, was less frequent in the King Vision VL group compared to the Linscope VL group. Similarly, the incidence of two adjustment maneuvers, involving OELM with external aids like stylets/bougie, was lower in the King Vision VL group than in the Linscope VL group. These observations suggest that the Linscope VL required more adjustment maneuvers than the King Vision VL. Possible explanations for the higher incidence of adjustment maneuvers in the Linscope VL group compared to the King Vision VL group include a less effective anti-fogging system, difficulties in maintaining hand-eye coordination, and challenges in passing the endotracheal tube through a stylet or bougie with the Linscope VL.

Assessment of the laryngeal view using the POGO score revealed comparable performance between the King Vision VL and Linscope VL, with the POGO score marginally higher for the Linscope VL. Both devices demonstrated effective glottis visualization during tracheal intubation procedures. Previous studies by Shimada et al. (2013) [12] and Murphy et al. (2014) [4] have reported superior POGO scores with the use of the King Vision VL compared to other VLs, suggesting its potential advantage in providing clearer views of the glottis. Contrary to these findings, our study observed similar POGO scores between the Linscope VL and King Vision VL. This discrepancy may be attributed to various factors, including differences in patient demographics, operator proficiency, and procedural techniques. Notably, the implementation of jaw thrust maneuvers in both study groups likely contributed to optimizing glottis visualization, thereby mitigating any discernible differences between the two devices. Furthermore, our study revealed that the majority of intubated patients were categorized as Grade I in terms of ease of intubation, with no significant difference noted between the two devices. This observation could be attributed to the predominance of patients classified as Mallampati class 1 and 2, indicating relatively straightforward airway management across both groups. While existing literature supports the superiority of the King Vision VL in terms of POGO scores, our findings highlight the importance of considering multiple factors that may influence these outcomes.

Based on the discussion above, it can be concluded that both the Linscope VL and King Vision VL demonstrated excellent performance in facilitating intubation among patients with no predicted difficult airway. These video laryngoscopes hold potential utility beyond routine intubation procedures, particularly in the evaluation of cut-throat injuries. Assessing parameters such as injury zone, depth, and anatomical location is crucial for informing appropriate management strategies in such cases [13]. While the Linscope VL is a relatively new device with limited available literature, our study found its intubation parameters to be comparable to those of the King Vision VL. This suggests that the Linscope VL may offer a viable alternative to established video laryngoscopes like the King Vision VL, particularly in settings where access to newer technologies is desired or required.

Overall, our study's findings support the efficacy and usability of both the Linscope VL and King Vision VL in clinical practice. Further research and clinical experience with the Linscope VL may provide additional insights into its performance and potential advantages in various clinical scenarios.

Limitations of the study

The present study has certain limitations. Firstly, blinding anesthesiologists to the devices was impractical due to the distinct shapes and sizes of the two instruments, potentially introducing observer bias. Secondly, the study exclusively involved elective general surgical patients belonging to ASA garde I and II only with no predicted difficult airway. Pediatric patients, geriatrics, and edentulous patients were not studied, limiting the applicability of results to emergency departments or other patient groups, including obstetrics, obese individuals, or those with cervical immobilization. Additionally, all intubations were performed by the same single operator. Therefore, it might not be appropriate to extrapolate the findings to such patient populations and generalised use.

## Conclusions

This randomized controlled trial compared the performance of Linscope and King Vision video laryngoscopes in tracheal intubation. King Vision VL demonstrated a shorter intubation time and significantly fewer optimization maneuvers compared to Linscope VL. Both devices can be considered practically equivalent for tracheal intubation. However, due to the well-established efficacy of King Vision VL supported by existing literature, it is suggested that King Vision be preferred over Linscope until further studies provide more evidence on its performance.
